# Electrocatalytic CO_2_ Reduction over Sn‐Cu Bimetallic Catalysts Synthesized via an Amalgamated Lithium Metal Method

**DOI:** 10.1002/advs.77199

**Published:** 2026-08-17

**Authors:** Minghao Xie, Shichen Guo, Jianxin Wang, Haozhe Zhang, Xun Zhan, Lauren Valentino, Di‐Jia Liu

**Affiliations:** ^1^ Chemical Sciences and Engineering Division Argonne National Laboratory Lemont Illinois United States; ^2^ Energy Systems and Infrastructure Assessment Division Argonne National Laboratory Lemont Illinois United States; ^3^ Pritzker School of Molecular Engineering The University of Chicago Chicago Illinois United States; ^4^ Walker Department of Mechanical Engineering The University of Texas at Austin Austin Texas United States; ^5^ Applied Materials Division Argonne National Laboratory Lemont Illinois United States

**Keywords:** acetaldehyde, amalgamated lithium metal, CO_2_RR, Sn‐Cu

## Abstract

Electrochemical carbon dioxide reduction reaction (eCO_2_RR) provides a pathway to convert CO_2_ into value‐added chemicals. Here, we report a family of carbon‐supported tin–copper (Sn–Cu) bimetallic catalysts synthesized via an amalgamated lithium metal (ALM) method with controlled tuning of metal dispersion from isolated atoms to nanocrystallites. As metal loadings increase, the catalyst structure evolves from atomically dispersed Sn‐Cu dual sites (Sn_1.5_Cu/C‐0.75) to Sn‐rich nanodomains (Sn_3_Cu/C‐13) and Cu/Sn crystallites (Sn_3_Cu/C‐24), leading to a systematic shift in CO_2_RR selectivity from acetaldehyde to formate at low overpotentials. Notably, Sn_1.5_Cu/C‐0.75 achieves a Faradaic efficiency of 49.7% for acetaldehyde at −0.5 V versus the reversible hydrogen electrode. The CO_2_ conversion pathways behind these selective, size‐modulated catalysts are elucidated by structural characterization, together with electrochemical analysis of structure‐selectivity correlations, establishing a clear structure‐property relationship for tuning CO_2_RR pathways.

## Graphical abstract

An amalgamated lithium metal method enables systematic control of Sn–Cu active‐site dimensions from isolated atoms to nanocrystallites. Increasing the metal loading shifts CO_2_ electroreduction selectivity from acetaldehyde on highly dispersed Sn–Cu sites to formate on Sn‐rich crystallites, revealing how bimetallic dispersion and local coordination govern competing multicarbon and C_1_ reaction pathways.

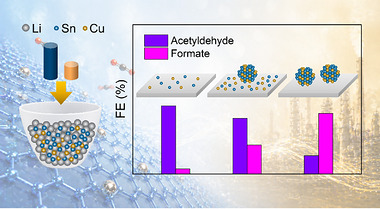

## 1 Introduction

Electrochemical CO_2_ reduction reaction (eCO_2_RR) enables the conversion of CO_2_ into a range of valuable chemicals through complex multi‐electron transfer pathways [1, 2]. However, controlling product distribution remains a central challenge. This conversion not only competes with the hydrogen evolution reaction (HER), but also involves multiple possible CO_2_ reduction pathways. Catalyst properties, such as electronic structure, surface coordination, and intermediate binding strength, play critical roles in determining eCO_2_RR outcomes [3, 4, 5]. Small variations in these parameters can significantly alter the reaction pathways, highlighting the importance of understanding the influences of catalyst design.

Bimetallic catalysts provide an effective platform to probe and tune the structure‐function relationships. Compared to mono‐metallic catalysts, incorporating a second metal could perturb both the geometric arrangement and electronic structure of the active sites. These changes could give rise to synergistic interactions that reshape reaction pathways, in part by relaxing conventional scaling relationships, where strengthening the adsorption of one intermediate typically leads to the proportional stabilization of others and thus constrains selectivity [6]. Since around 2014, bimetallic catalysts for eCO_2_RR have been extensively explored through variations in composition and synthesis methods, offering versatile approaches to modulate catalytic behavior [7]. Recent developments in synthesis methods have introduced new opportunities to control catalyst structure at fine length dimensions, down to the nanoscale and even the atomic scale. Among these, amalgamated lithium metal (ALM) methods have emerged as a versatile tool for preparing mono‐metallic catalysts, enabling the synthesis of eCO_2_RR catalysts ranging from single atom dispersions to ultrasmall metallic crystallites [8, 9]. This approach starts by dissolving catalyst metal in molten lithium (Li), followed by quenching the liquid lithium‐metal amalgam at room temperature to “freeze” the metal in a uniformly dispersed state. The Li is then converted to lithium hydroxide (LiOH) by humidification, and the uniformly dispersed metal is transferred to supports such as high surface area carbon, all at room temperature. Since this is a physical process, transferring metal to support at ambient temperature could circumvent the drawback of chemical synthesis, in which reducing precursors at elevated temperature often causes uncontrollable agglomeration of metallic catalytic centers. This method is theoretically applicable to a wide range of metals on the basis of lithium amalgam metallurgy, and several resulting catalysts have demonstrated distinct selectivity, such as ethanol formation on copper (Cu)‐based systems [8], and acetate, ethanol, and formate on Sn‐based systems [9].

In this work, we demonstrate the first application of the ALM method to bimetallic catalyst synthesis for Sn‐Cu catalysts. Individually, Cu and Sn are both known to promote multi‐carbon (C_2+_) products and formate (HCOO^−^) formation during eCO_2_RR [10, 11], while Sn‐Cu bimetallic systems have been reported to exhibit tunable selectivity, primarily between carbon monoxide (CO) and formate [12, 13, 14, 15]. In this study on bimetallic Sn‐Cu catalysts, we observed a relatively high eCO_2_RR Faradic efficiency (FE) of 49.7% for acetaldehyde at a low metal loading of 0.75 wt.%,. With an increase in both Sn and Cu loadings in the catalyst by varying the amount of dissolved metal during the ALM process, the main CO_2_ conversion products shift from acetaldehyde to formate with FE of 63.6% under high metal loading of 24 wt.%. Accompanying metal loadings increase, significant changes in catalyst structure and composition, from single segregated Sn and Cu atoms to Sn‐Cu bimetallic clusters, were also found, providing new understanding on the structure property‐performance relationships.

## 2 Results and Discussion

### 2.1 Synthesis of Sn‐Cu Bimetallic Catalysts by ALM

**FIGURE 1:**
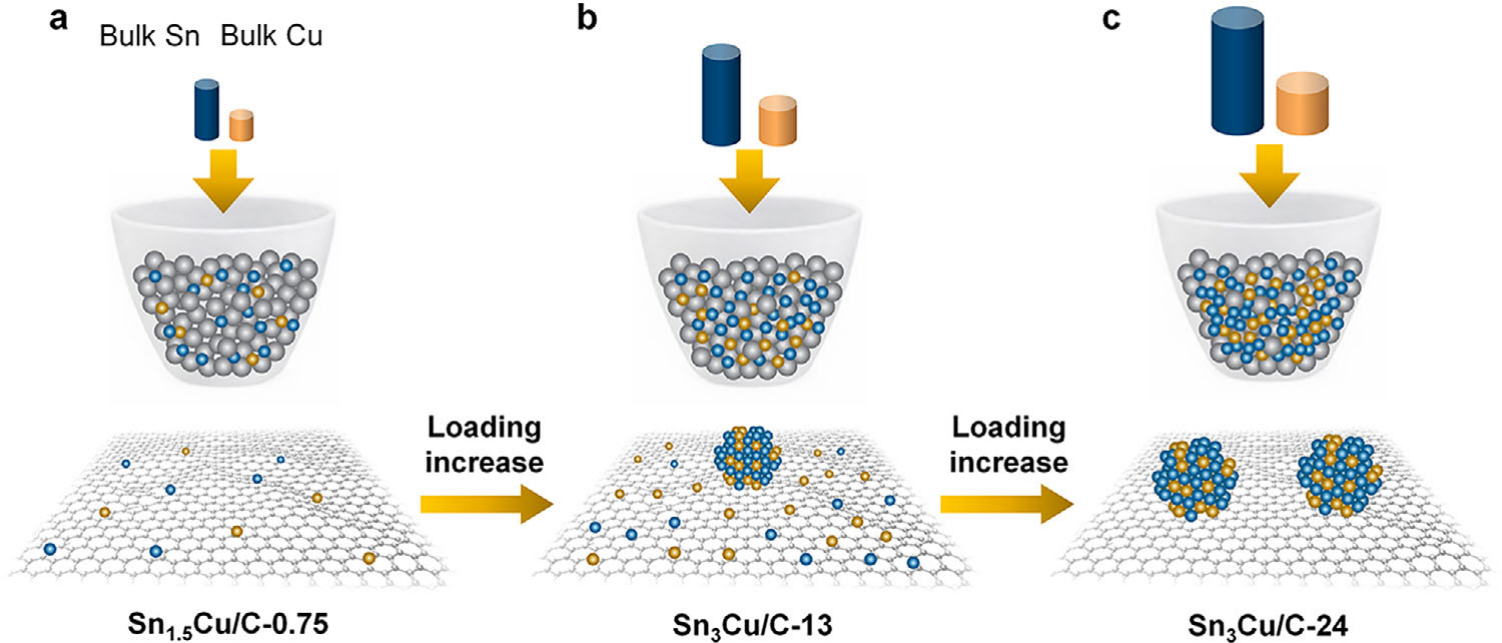
Syntheses of Sn‐Cu/C catalysts with different metal dispersion using the ALM method, leading to catalysts from (a) isolated atoms of Sn and Cu, (b) a combination of single atoms with nanoparticles, and (c) particles only over the surface of carbon supports.

Our previous studies revealed that monometallic Cu or Sn‐based single‐atom catalysts prepared by the ALM method led to high selectivity toward ethanol and acetate formations [8, 9]. In this report, we extended ALM synthesis to the bimetallic system and fabricated a set of carbon‐supported Sn–Cu catalysts. To understand the impact of increasing bimetallic catalyst loading on eCO_2_RR products, we varied Sn and Cu dispersion and active site dimensions by introducing different quantities of metallic Sn and Cu into molten Li during the amalgamation stage (Figure 1). Both Sn and Cu were homogeneously dissolved in the molten Li and remained uniformly distributed across all loadings after rapidly quenching into a solid state at room temperature. Following the transformation of Li into LiOH in humidified air, the resulting powder mixtures were combined with carbon black, and LiOH was subsequently removed through water washing, thereby depositing Sn and Cu onto the carbon surface. Throughout this transfer process, additional hydroxyl and carboxyl functionalities were generated as a result of a strongly alkaline reaction to the carbon matrix [9], which serve as the anchoring sites of highly dispersed Sn and Cu species on carbon based on our established ALM studies [9]. All procedures conducted after the quenching step were carried out at ambient temperature through physical mixing. Such a process minimizes unevenness of metal particle size distribution and avoids heat‐induced agglomeration. This unique characteristic differs from the conventional binary catalyst synthesis routes through wet chemistry that rely on chemical precursors followed by high‐temperature reduction, which frequently leads to uncontrolled metal particle growth and broad size distributions due to agglomeration.

Three supported catalysts with total metal loadings of 0.75 wt.%, 13 wt.%, and 24 wt.% and corresponding Sn:Cu atomic ratios of 1.5, 3.0, and 2.8 were prepared. The actual loadings of Sn and Cu were measured by X‐ray fluorescence (XRF) (Figure S1) and are summarized in Table S1. These catalysts are labeled based on their measured metal loadings and atomic ratios. For example, the catalyst with a measured metal loading of 0.75 wt.% and Sn:Cu ratio of 1.5 is named Sn_1.5_Cu/C‐0.75. For clarification, the catalysts with measured metal loadings of 13 wt.% and 24 wt.% and corresponding Sn:Cu ratios of 3.0 and 2.8 are named as Sn_3_Cu/C‐13 and Sn_3_Cu/C‐24, respectively.

### 2.2 Characterization of Electrocatalysts

**FIGURE 2:**
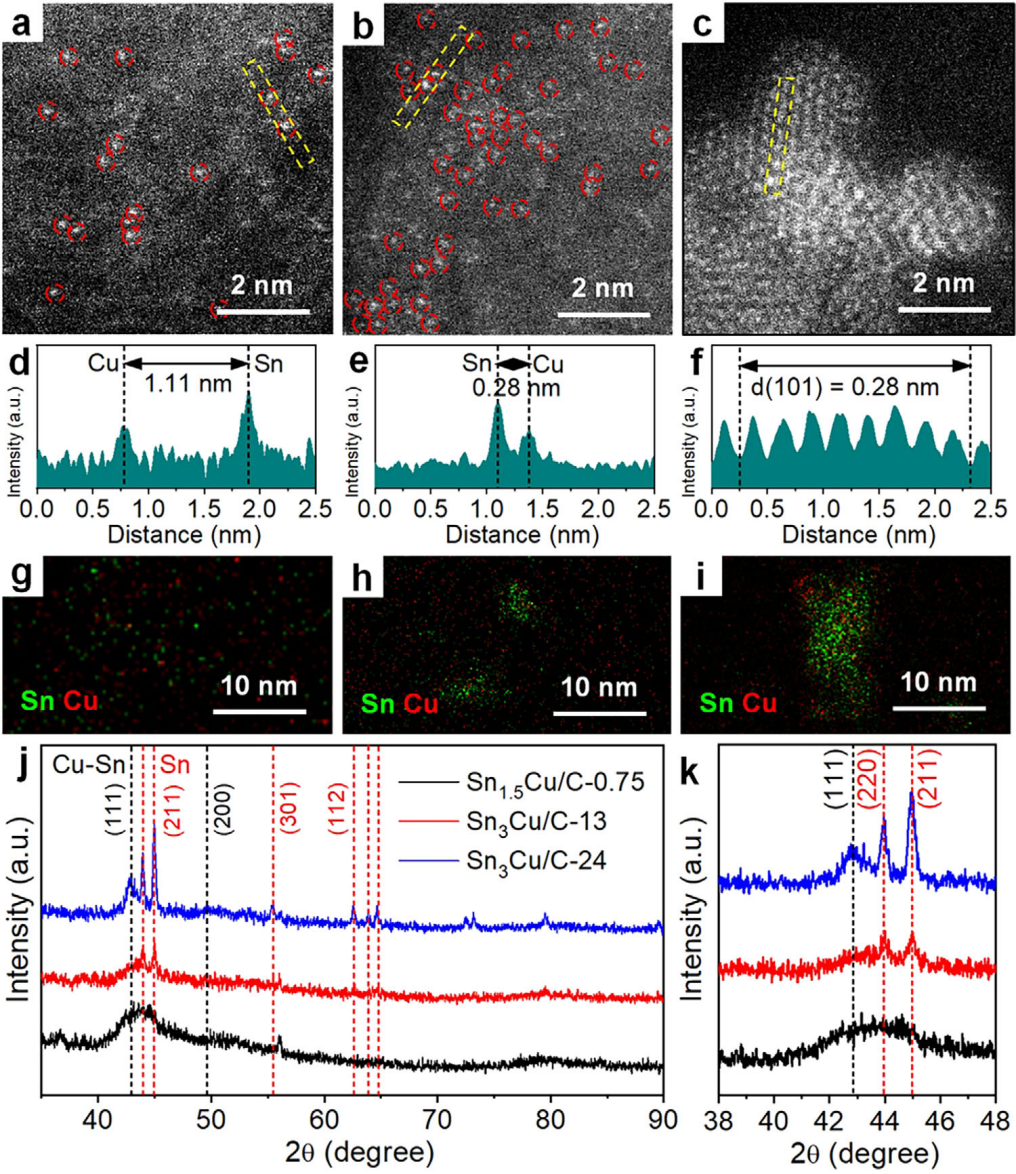
HAADF‐STEM images of (a) Sn_1.5_Cu/C‐0.75, (b) Sn_3_Cu/C‐13 and (c) Sn_3_Cu/C‐24, showing the transition from the isolated Sn and Cu atoms (in red circles) to isolated atoms with increased density, and to Sn‐Cu bimetallic nanodomains. (d) Intensity profile of single atomic columns following the yellow box in panel (a), showing the distance between Sn and Cu atoms. (e) Intensity profile of single atomic columns following the yellow box in panel (b), demonstrating a distance shrinkage between Sn and Cu atoms. (f) Intensity profile of single atomic columns following the yellow box in panel (c), demonstrating the lattice spacing of Sn‐Cu bimetallic nanodomains. Elemental mapping of (g) Sn_1.5_Cu/C‐0.75, (h) Sn_3_Cu/C‐13 and (i) Sn_3_Cu/C‐24, showing the migration from isolated Sn and Cu atoms to Sn‐Cu bimetallic nanodomains with increased metal loading. (j, k) XRD patterns of catalysts. Red dashed lines: JCPDS #04‐ 0673 (Sn). Black dashed lines: JCPDS #65‐7047 (Cu_41_Sn_11_), demonstrating the presence of Sn and Sn‐Cu nanocrystallites with increased metal loadings.

At the lowest loading of 0.75 wt.%, the Sn and Cu in Sn_1.5_Cu/C‐0.75 are in a highly dispersed atomistic form, as shown by a high‐angle annular dark‐field aberration‐corrected scanning transmission electron microscopy (HAADF‐STEM) (Figure 2a; Figure S2a). The intensity profile obtained from a line scan across the yellow box over isolated atoms in Figure 2a shows individual peaks with distinguishable intensity (Figure 2d), demonstrating the presence of Cu and Sn with different atomic numbers and thus brightness under STEM. A representative distance measured between the Cu and Sn atoms is 1.11 nm. Energy dispersive X‐ray spectroscopy (EDS) elemental mapping further verifies the uniform dispersion of both Sn and Cu atoms on the surface of a carbon support (Figure 2g; Figure S2d). As the loading increased to over 17‐fold to 13 wt.%, Sn and Cu atoms in Sn_3_Cu/C‐13 started to assemble into crystallites (Figure S2b), while atomically dispersed species still exist with higher density over the surface of the carbon support (Figure 2b). The intensity profile reveals a reduced distance between Cu and Sn atoms with a value of 0.28 nm, close to the reported Cu─Sn bonds in the alloy phase, suggesting the formation of metallic bonds between Sn and Cu atoms [16, 17]. EDS elemental mapping proves the nanodomains are composed of Sn, demonstrating the partial agglomeration of Sn as the metal loading increased (Figure 2h; Figure S2e). Further increasing the metal loading by almost another 2‐fold to 24 wt.% in Sn_3_Cu/C‐24 leads to the formation of 5 nm to 10 nm crystallites with nanodomains in Sn_3_Cu/C‐24, and isolated surface atoms are no longer observed (Figure 2c; Figure S2c). The intensity profile reveals a lattice spacing of 0.28 nm, corresponding to the distance between [101] planes of β‐Sn crystallite [18, 19]. EDS elemental mapping demonstrates that the crystallite is composed of Sn with a slight amount of Cu on the surface, demonstrating the agglomeration between Sn and Cu as the metal loading further increased (Figure 2i; Figure S2f).

Powder X‐ray diffraction (PXRD) detected no scattering peak from Sn‐ and Cu‐related species in Sn_1.5_Cu/C‐0.75 (Figure 2j,k; Figure S3), consistent with the highly dispersed isolated active metal sites in the absence of a long‐range crystal structure in the catalyst. In contrast, the β‐Sn phase was observed in Sn_3_Cu/C‐13, while no scattering peak from Cu‐related species was observed, indicating the formation of large crystallites of Sn, with isolated Cu and Sn species remaining as the metal loading increased. This result is understandable because the loading of Sn is 3 times higher than Cu and thus causes Sn agglomeration. When further increase the metal loading to 24 wt.%, a new peak with low crystallinity assigned to Cu‐Sn phase appeared, unveiling alloy of Sn and Cu into the crystallites with distinctive phases [20].

Cu K‐edge X‐ray absorption spectroscopy was employed to investigate the electronic and local coordination structures of Cu in Sn_3_Cu/C‐13. The edge feature of the X‐ray absorption near edge structure (XANES) spectrum of Sn_3_Cu/C‐13 resembles the intermediate dominated by CuO with a minor contribution by Cu, indicating the coexistence of metallic and partially oxidized Cu species (Figure S4a). The Fourier‐transformed extended X‐ray absorption fine structure (EXAFS) spectrum displays contributions from both the Cu‐O and metallic coordination shells (Figure S4b). Quantitative EXAFS fitting in both *R* and *k* spaces shows good agreement with the experimental data (Figure S5), yielding Cu─O, Cu─Cu, and Cu─Sn coordination paths at 1.902 ± 0.009, 2.563 ± 0.010, and 2.731 ± 0.031 Å, with corresponding coordination numbers of 2.15 ± 0.08, 2.57 ± 0.14, and 2.90 ± 0.18, respectively (Table S2). The simultaneous presence of Cu─Cu and Cu─Sn coordination confirms the formation of undercoordinated Sn─Cu bimetallic domains, whereas the Cu─O contribution originates from oxygen‐containing functional groups on the carbon support that anchor the dispersed Cu species. These results are consistent with the coexistence of isolated metal sites and nanoscale Sn─Cu domains observed by electron microscopy.

### 2.3 eCO_2_RR Measurement

**FIGURE 3:**
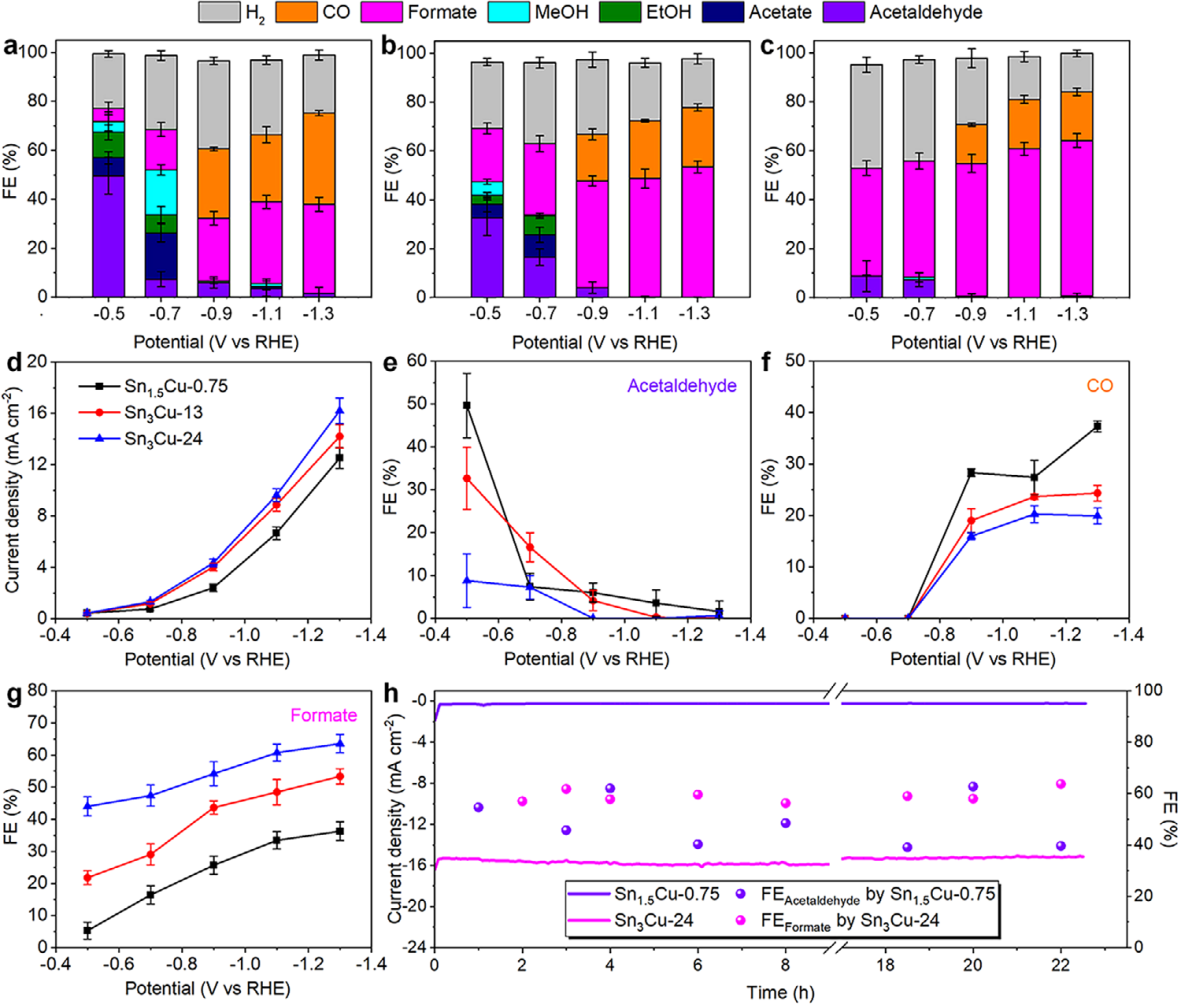
Electrocatalytic CO_2_RR measurement. FEs and the product distributions under different polarization potentials over (a) Sn_1.5_Cu/C‐0.75, (b) Sn_3_Cu/C‐13, and (c) Sn_3_Cu/C‐24. (d) Geometric current densities and FEs under different polarization potentials toward (e) acetaldehyde, (f) CO, and (g) formate formation. The data were averaged over three repeated measurements with the standard deviations marked by error bars. (h) FEs for acetaldehyde and formate formation and their total current density for Sn_1.5_Cu/C‐0.75 and Sn_3_Cu/C‐24 as a function of time during the chronoamperometric electrolysis at −0.5 V and −1.3 V vs. RHE, respectively. The stability testing conditions were selected based on respective optimal conditions for acetaldehyde and formate production.

The eCO_2_RR performance of three Sn─Cu bimetallic catalysts was evaluated in 0.1 m KHCO_3_ under different cell potentials to illustrate how site structures altered the selectivity (Figure 3a–c). The experimental details are presented in the Experimental Section. Figure 3d shows total current densities at applied potentials from −0.5 V to −1.3 V vs. reversible hydrogen electrode (RHE) for the catalysts. The current density increases systematically with the metal loading, indicating that catalytic activity scales with the amount of deposited metal, as the metallic phase constitutes the primary reaction domain. Figure 3a–c presents Faradaic efficiency (FEs) distributions of the key eCO_2_RR products over the three catalysts. The FEs of selected key products (acetaldehyde, CO, and formate) as a function of cell potential are summarized in Figure 3e–g.

When comparing catalysts with different metal loadings, distinct product distributions are observed. For the Sn_1.5_Cu/C‐0.75 sample, where isolated single‐atom sites dominate, C_2+_ products are favored at low overpotential, with C_2+_ FE reaching 67.4% dominated by acetaldehyde with FE of 49.7% at −0.5 V vs. RHE (Figure 3a,e), which is detected by its hydrated form by nuclear magnetic resonance (NMR) spectroscopy (Figure S6). It is noteworthy that the distinct selectivity of Sn_1.5_Cu/C‐0.75 toward acetaldehyde, compared with the known ethanol‐ and acetate‐oriented behavior of the monometallic ALM‐derived Cu and Sn systems [8, 9], supports the role of coupled Sn–Cu sites rather than carbon alone. Previous studies demonstrate that the carbon support serves mainly as the high‐surface‐area matrix for dispersing the metal species and is not assigned as the origin of the observed acetaldehyde selectivity [8, 9]. For the Sn_3_Cu/C‐24 sample, where both Sn particles and Sn–Cu particles are present, formate dominates the product distribution across all applied potentials, reaching 63.6% at −1.3 V vs. RHE (Figure 3g), with only minor contributions from C_2+_ and CO. The Sn_3_Cu/C‐13 sample contains both single atoms and nanocrystalline domains. Its eCO_2_RR product distribution corresponds to an intermediate case, with C_2+_ and formate selectivity falling between those of Sn_1.5_Cu/C‐0.75 and Sn_3_Cu/C‐24, reflecting the coexistence of two types of distinct active sites of different catalytic conversion preferences.

eCO_2_RR product distributions are also sensitive to operating cell potential. As the cathodic potential increased from −0.5 V to −1.3 V vs. RHE, the FEs of C_2+_ chemicals decreased while the FEs of CO and formate increased. This trend suggests that milder overpotentials favor pathways involving extended proton‐electron transfers. Notably, CO formation shows a clear onset at approximately −0.9 V vs. RHE, accompanied by continuously diminishing C_2+_ production beyond that point. As both processes share the same ^*^CO intermediate at higher overpotentials [21], the observation suggests that the accelerated turnover rates with the increase of cell potential promote the desorption of the C_1_ intermediate product, interrupting C–C coupling in C_2+_ chemical formation during long and extended steps of proton‐coupled electron transfers [22].

The stability of the Sn‐Cu catalysts was further evaluated for Sn_1.5_Cu/C‐0.75 and Sn_3_Cu/C‐24 catalysts under their respective optimal conditions for acetaldehyde and formate production. During the chronoamperometric measurement over an extended period of 22 h, Sn_1.5_Cu/C‐0.75 maintained a stable current of approximately −0.23 mA cm^−2^ at −0.5 V vs. RHE, while Sn_3_Cu/C‐24 sustained a stable current of approximately −15.7 mA cm^−2^ at −1.3 V vs. RHE. (Figure 3h). For product selectivity, Sn_1.5_Cu/C‐0.75 exhibited an acetaldehyde FE of 49% with around 10% fluctuation over 22 h. The moderate variation in acetaldehyde FE during extended electrolysis can be attributed to the combined effects of low acetaldehyde concentration generated over Sn_1.5_Cu/C‐0.75 at the low current density and the intrinsic volatility of acetaldehyde during prolonged electrolysis [23, 24, 25]. The chronoamperometric current remains steady over 22 h, which argues against the assumption that severe catalyst deactivation is the primary cause of the fluctuation. Besides, Sn_3_Cu/C‐24 demonstrated stable formate selectivity throughout the 22 h test, maintaining a FE of 59% with around 3% fluctuation over 22 h (Figure 3h). Overall, the stable current response and sustained product selectivity demonstrate the good stability of the Sn‐Cu catalysts under electrochemical operations.

The large nanocrystals in Sn_3_Cu/C‐24 preferentially promote the formation of formate. This behavior is consistent with previous reports on Sn‐based and Sn‐Cu catalysts [12, 26], where Sn‐rich surfaces favor stabilization of the ^*^HCOO intermediate over ^*^CO. In contrast, the predominantly single‐atom Sn and Cu sites in Sn_1.5_Cu/C‐0.75 exhibit relatively high selectivity toward acetaldehyde production. Notably, acetaldehyde has been reported in several previous studies [23, 25, 27, 28], with the highest FE reaching 92% on Cu‐cluster catalysts and 88% on Cu‐Ag systems [23, 29]. Its formation is generally proposed to follow the ethanol pathway, terminating at the ^*^CH_3_CHO intermediate before further hydrogenation to ethanol [25]. For example, Jaramillo and coworkers reported that Ag incorporation into CuAg catalysts increases the energy barrier for the hydrogenation of *CH_3_CHO toward ^*^CH_3_CH_2_O [28], thereby promoting acetaldehyde desorption. However, because the present system is dominated by isolated single‐atom sites rather than metallic ensembles or clusters, the reaction mechanism and intermediate stabilization behavior are expected to differ substantially from those reported for conventional nanoparticle‐based catalysts.

**FIGURE 4:**
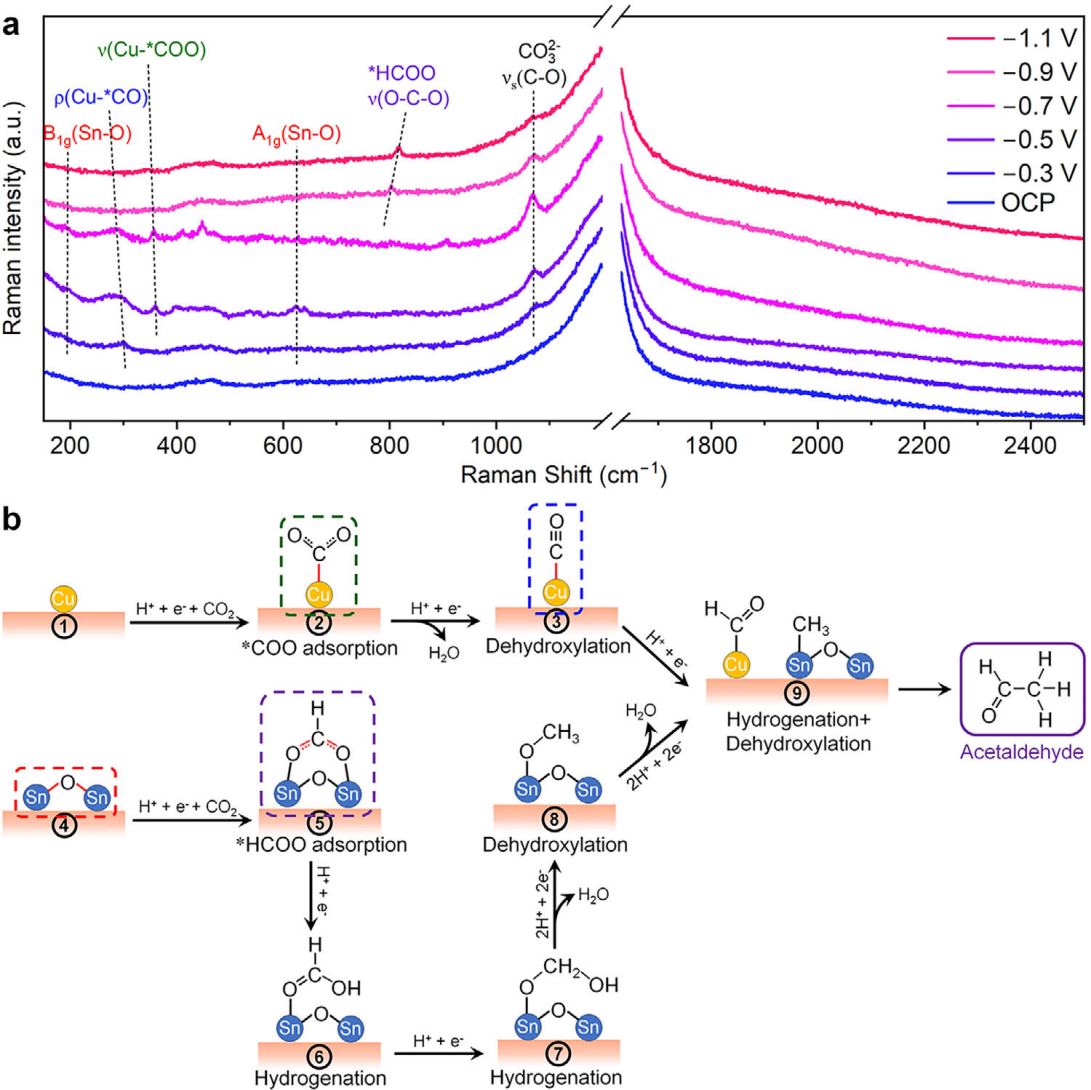
Reaction mechanism study. (a) Raman spectra of Sn_1.5_Cu/C‐0.75 in CO_2_‐saturated 0.1 m KHCO_3_ during stepwise change from OCP to −1.1 V vs. RHE. The detailed assignments and references can be found in Table S3. (b) Proposed reaction mechanism with nine states for the formation of acetaldehyde over Sn_1.5_Cu/C‐0.75. The experimentally observed intermediates are highlighted in dashed boxes.

To identify the surface intermediates associated with acetaldehyde formation, in situ surface‐enhanced Raman spectroscopy (SERS) was performed on Sn_1.5_Cu/C‐0.75 in CO_2_‐saturated 0.1 m KHCO_3_ while the potential was stepped from the open‐circuit potential (OCP) to −1.1 V vs. RHE (Figure 4a; Table S3). Due to strong interference from D/G bands of the carbon support and ultralow catalyst metal loadings, we could only obtain the spectroscopic features between 200 cm^−1^ and 1100 cm^−1^. At the open‐circuit potential (OCP), only the vibrational features of the carbon support are evident. From the OCP up to −1.1 V vs. RHE during eCO_2_RR, a peak at 1068.2 cm^−1^ was observed without frequency shift, which is associated with CO_3_^2−^ from the deprotonation of HCO_3_^−^ near the catalyst surface [30, 31]. The onset of CO_2_ reduction at −0.3 V is accompanied by a band at 285.4 cm^−1^ – 297.4 cm^−1^ assigned to the frustrated rotation/copper‐carbon vibration of Cu‐^*^CO [31]. This band progressively red‐shifts as the potential is swept from −0.3 V to −0.7 V, revealing a negative vibrational Stark effect and stabilization of the intermediate through the alignment of the electric field and the dipole moment of surface‐bound ^*^CO. A second potential‐sensitive feature at 347.8 cm^−1^ – 358.8 cm^−1^, assigned to the Cu–C stretching mode of the carboxylate‐like Cu‐^*^COO intermediate [31], is most apparent between −0.3 V and −0.5 V and likewise red‐shifts with increasing cathodic bias. In contrast, the ^*^HCOO band at 800.7 cm^−1^–817.4 cm^−1^ becomes clearly resolved from −0.9 V to −1.1 V and blue‐shifts with potential, corresponding to a positive Stark response [32]. The opposite Stark directions of Cu‐^*^CO/Cu‐^*^COO and ^*^HCOO indicate distinct adsorption geometries and dipole orientations within the interfacial electric field, while their coexistence demonstrates that CO_2_ is activated concurrently through carbon‐bound ^*^COOH/^*^CO and oxygen‐bound ^*^HCOO pathways. Two potential‐insensitive bands at approximately 194 cm^−1^ and 627 cm^−1^ are assigned to the asymmetric stretching (B_1g_) and symmetric stretching (A_1g_) of Sn─O bond [33, 34]. Their absence of a Stark response distinguishes these structural Sn─O modes from adsorbate vibrations and is consistent with a previous ALM‐derived Sn study where Sn single atoms can form oxidized ultrasmall clusters under eCO_2_RR conditions [9].

These spectroscopic observations support the dual‐branch mechanism depicted in Figure 4b. In the upper branch, one CO_2_ molecule is activated at a Cu‐containing site through a carboxyl intermediate (^*^COO, State 1–2), followed by dehydroxylation to ^*^CO (State 3) and hydrogenation to ^*^CHO (State 9). In parallel, a second CO_2_ molecule adsorbs through a bidentate ^*^HCOO configuration (State 4–5) at the neighboring oxidized Sn clusters (dedicated as µ‐oxo Sn‐O‐Sn) and undergoes sequential proton‐coupled electron transfers through oxygenated C_1_ intermediates (States 6–8), ultimately yielding a surface ^*^CH_3_ species after dehydroxylation (State 9). The simultaneous SERS detection of Cu‐^*^COO, Cu‐^*^CO, and ^*^HCOO directly captures the early intermediates of these two parallel trajectories. The proximity of isolated Sn and Cu sites then enables cross‐coupling between the carbonyl‐derived ^*^CHO fragment and the deeply hydrogenated ^*^CH_3_ fragment, producing acetaldehyde. This pathway rationalizes why atomically dispersed Sn‐Cu sites favor acetaldehyde at mild potentials: the dual‐site environment retains two chemically distinct C_1_ intermediates long enough to complete multistep proton‐electron transfer and selective C─C bond formation, whereas increasingly cathodic potentials accelerate the shorter CO‐ and formate‐producing branches and reduce acetaldehyde selectivity.

## 3 Conclusion

We demonstrated that the paths for the electrochemical conversion of CO_2_ to organics, especially chemicals such as acetaldehyde with FE of 49.7% and formate with FE of 63.6%, can be modulated by the dimension of Sn and Cu active sites with relatively high selectivity and low onset potentials. The new ALM synthesis offered a new low‐temperature method of preparing bimetallic catalysts such as Sn‐Cu binary catalysts with tunable active center size. Such flexibility could lead to changes in structure‐property correlations between FE, catalyst loading, and operating potential, and their transitions from one to another. Advanced structural characterization revealed the isolated nature of the catalyst site. Furthermore, we proposed a reaction mechanism for the formation of acetaldehyde over the Sn‐Cu dual‐site catalyst, in which CO_2_ is reduced via a ^*^HCOO pathway to generate ^*^CH_3_ on Cu sites, while a second CO_2_ is activated through a ^*^COOH pathway on Sn sites to form ^*^CHO. This is followed by substantial coupling to produce acetaldehyde, with competing intermediates enabling acetate and ethanol formation as detected. The direct conversion of CO_2_ to value‐added organics with high FE and low overpotential offers a promising pathway for utilizing CO_2_ as the feedstock in chemical manufacturing.

## 4 Experimental Section

### 4.1 Synthesis of Carbon‐Supported Sn‐Cu Catalysts

Synthesis of Sn‐Cu bimetallic catalysts supported on carbon was conducted in an Ar‐filled glovebox (oxygen level <0.3 ppm) similar to previously reported methods []. 0.29 mol (2.0 g) of Li metal (99.9%, Sigma‐Aldrich) was added into a nickel crucible and heated to 320 °C to obtain molten Li with a mirror‐like surface. Predetermined amounts of Cu wire and Sn foil (99.9%, Alfa Aesar) were then introduced into the molten Li. For Sn_1.5_Cu/C‐0.75, Sn_3_Cu/C‐13 and Sn_3_Cu/C‐24, the Cu/Sn loadings were 0.095 mmol (6.05 mg) / 0.285 mmol (33.8 mg), 1.587 mmol (100.8 mg) / 4.762 mmol (565.2 mg), and 3.337 mmol (211.88 mg) / 10.01 mmol (1.188 g), respectively. An ultrasonic homogenizer was applied during the process to promote uniform dispersion of Sn and Cu within the molten Li, facilitating atomic‐level mixing while suppressing phase segregation, precipitation, and settling. The system was maintained at 320 °C for 2 h, during which the molten solution retained a mirror‐like surface. The molten metal was then rapidly poured onto a clean 316 stainless steel plate to quench and form a solid solution. After cooling, the resulting Sn‐Cu‐Li solid was removed from the glovebox, sectioned into small pieces, and exposed to humidified air in a desiccator at ambient temperature to gradually convert Li to LiOH. After approximately two weeks, the obtained Sn‐Cu‐LiOH composite was thoroughly mixed with 2.5 g of carbon black (Ketjenblack EC‐300J, Fuel Cell Store) via ball milling. The resulting mixture was subsequently washed with excess deionized water to leach off LiOH, followed by filtration. The final catalyst was dried under vacuum at 60 °C for 24 h prior to use.

### 4.2 Material Characterizations

Powder XRD measurements were carried out on a Rigaku Miniflex diffractometer with Cu Kα radiation (*λ* = 1.5406 Å). Samples were prepared by placing them on a silicon zero diffraction plate with amorphous carbon‐based grease. Catalysts were visualized using HAADF‐STEM at the University of Illinois, Chicago, which is a probe‐corrected JEOL JEMARM200CF equipped with a 200‐kV cold‐field emission gun. This STEM is also equipped with an EDX spectroscopy analyzer (Oxford EDX XMAX80 system). The samples were prepared by using ultra‐thin carbon film‐covered gold transmission electron microscopy grids (TED PELLA, Inc.) to collect the samples dispersed in ethanol under sonication. The gold grids were then dried at 60 °C. A JEOL IT800HL scanning electron microscope operated at 20 kV was also used to prescreen the samples to check elemental distribution. The weight percentages of Sn and Cu catalysts were measured by X‐ray fluorescence (XRF) analysis (NEX CG Energy Dispersive X‐ray Fluorescence Spectrometer). To accurately measure the Sn loading, the fluorescence signals at the Sn K‐edge and Cu K‐edge were collected from (SnCl_2_·2H_2_O+CuSO_4_·2.5H_2_O)/carbon samples with varying concentrations and used to generate a calibration curve. The catalysts were subsequently analyzed by comparing the XRF signal intensity with the calibration curve. XAS involving operando XANES and EXAFS was performed at 20‐BM (beamlines) of the Advanced Photon Source (APS) at the Argonne National Laboratory to investigate the local environment around the Cu atoms of the samples. The XANES spectroscopy was performed in fluorescence mode due to the low loading of Cu on carbon at the Cu *k* edge (9,659 eV). The Cu foil EXAFS was measured for energy calibration for each scan of the samples. For each sample, several scans were taken and averaged to gain a better signal‐to‐noise ratio.

### 4.3 Electrode Preparation

Catalyst ink was prepared by dispersing 5 mg of the as‐synthesized catalyst in 1 mL of methanol, followed by adding 54 µL of Nafion solution (5 wt%, Sigma‐Aldrich), corresponding to a catalyst‐to‐Nafion mass ratio of 1:2. The suspension was sonicated for 1 h to obtain a homogeneous ink. Subsequently, 50 µL of the ink was drop‐cast onto a 1 cm × 1 cm carbon paper substrate (GDL340, Fuel Cell Store) and dried under an infrared lamp. This deposition step was repeated four times to achieve a total catalyst loading of 1 mg cm^−2^. The prepared electrode was then dried under vacuum at 60°C for 24 h prior to use.

### 4.4 Electrochemical Measurements

Electrochemical measurements were carried out in a gas‐tight H‐type cell. The as‐prepared electrode served as the working electrode, while a gold wire and an Ag/AgCl electrode were used as the counter and reference electrodes, respectively. The reference electrode was calibrated against RHE in 0.1 m KHCO_3_ prior to use. To perform the reaction, both compartments were filled with 30 mL of 0.1 m KHCO_3_ electrolyte. CO_2_ was continuously introduced into the working chamber at a flow rate of 30 mL min^−1^. The electrolysis was run for 30 min using a potentiostat (Biologic SP‐150) at constant voltage. The outlet gas stream from the working compartment was directly analyzed using an online gas chromatograph (Agilent 7890B). The GC inlet flow rate was corrected using a mass flow meter pre‐calibrated with pure CO_2_. Liquid products were quantified from the electrolytes in both compartments by nuclear magnetic resonance spectroscopy (NMR, Bruker 500 MHz) using DMSO and phenol in D_2_O as standard. Each test was performed at least three times under the same conditions.

### 4.5 In Situ Raman Spectrum Measurements

In situ electrochemical surface‐enhanced Raman spectroscopy (SERS) measurements were performed using a Renishaw confocal Raman spectrometer equipped with a 785 nm diode laser and a 1,200 grooves mm^−1^ grating. The laser power density at the sample surface was approximately 0.1 mW µm^−2^. Raman spectra were collected using a 40× Nikon water‐immersion/dipping objective (NA = 0.8; working distance = 3.5 mm), with an integration time of 30 s. The spectrometer was calibrated using a Si standard before measurement. To prepare the working electrode, 5 mg of catalyst was dispersed in 1 mL of methanol and sonicated for 1 h. An aliquot of the catalyst ink (40 µL) was mixed with 20 µL of an aqueous suspension of SERS‐active Au@SiO_2_ nanorods and deposited onto carbon paper with a geometric area of 0.2 cm^2^, followed by vacuum drying overnight. Measurements were conducted in a PEEK spectroelectrochemical cell using the catalyst‐coated carbon paper, a Pt wire, and an Ag/AgCl electrode as the working, counter, and reference electrodes, respectively. CO_2_‐saturated 0.1 m KHCO_3_ (20 mL) was used as the electrolyte, with CO_2_ continuously supplied at 20 mL min^−1^. Before spectral acquisition, each potential was held for 1 min to stabilize the electrode surface.

## Author Contributions

**Minghao Xie**: investigation, formal analysis, writing – original draft. **Shichen Guo**: investigation, formal analysis, writing – original draft. **Jianxin Wang**: investigation. **Haozhe Zhang**: investigation. **Xun Zhan**: investigation, data curation. **Lauren Valentino**: investigation, writing – review and editing, funding acquisition. **Di‐Jia Liu**: conceptualization, investigation, supervision, funding acquisition, writing – review and editing.

## Conflicts of Interest

The authors declare no conflict of interest.

## Supporting information




**Supporting File**: advs77199‐sup‐0001‐SuppMat.docx.

## Data Availability

The data that support the findings of this study are available from the corresponding author upon reasonable request.
