# Supplementary material for: Electrocatalytic CO2 Reduction over Sn‐Cu Bimetallic Catalysts Synthesized via an Amalgamated Lithium Metal Method
Source: Adv Sci (Weinh). 2026 Aug 17:e77199. Online ahead of print. doi: 10.1002/advs.77199 (PMC13479848; doi:10.1002/advs.77199)
Supplement: Supplementary file 1 [file ADVS-9999-e77199-s001.docx]

Supplementary Information

**Electrocatalytic CO_2_ reduction over Sn-Cu bimetallic catalysts synthesized via an** **amalgamated lithium metal method**

Minghao Xie,^1,6^ Shichen Guo,^2,6^ Jianxin Wang,^1^ Haozhe Zhang,^1,3^ Xun Zhan,^4^ Lauren Valentino,^5^ and Di-Jia Liu^1,3*^

1. Chemical Sciences and Engineering Division, Argonne National Laboratory, Lemont, Illinois 60439, United States

2. Energy Systems and Infrastructure Assessment Division, Argonne National Laboratory, Lemont, Illinois 60439, United States

3. Pritzker School of Molecular Engineering, The University of Chicago, Chicago, Illinois 60637, United States

4. Walker Department of Mechanical Engineering, The University of Texas at Austin, Austin, Texas 78712, United States

5. Applied Materials Division, Argonne National Laboratory, Lemont, Illinois 60439, United States

6. These authors contributed equally to this work: Minghao Xie, Shichen Guo

*Corresponding E-mail: djliu@anl.gov

**Figure S1.** XRF calibration curve for measurement of metal loading of (a) Sn and (b) Cu for Sn_1.5_Cu/C-0.75 (black star), Sn_3_Cu/C-13 (red star), Sn_3_Cu/C-24 (blue star).

**Figure S2.** Low magnification HAADF-STEM images of (a) Sn_1.5_Cu/C-0.75, (b) Sn_3_Cu/C-13 and (c) Sn_3_Cu/C-24, showing the generation of nanocrystallites with increased metal loading. Low magnification elemental mapping of (d) Sn_1.5_Cu/C-0.75, (e) Sn_3_Cu/C-13 and (f) Sn_3_Cu/C-24, showing the dispersion uniformity of Sn and Cu over different metal loading.

**Figure S3.** XRD patterns of catalysts. Red dashed lines: JCPDS # 04-0673 (Sn). Black dashed lines: JCPDS #65-7047 (Cu_41_Sn_11_).

**Figure S4.** (a) Cu *k*-edge normalized XANES spectra and (b) Fourier transform of *k*^2^-weighted *χ* function in R space EXAFS data of Sn_3_Cu/C-13 plus Cu foil and CuO as references.

**Figure S5.** (a) Fitting results of the Cu *k*-edge R-space EXAFS spectrum of Sn_3_Cu/C-13 and (b) EXAFS fitting (red) in k-space vs. the experimental data (blue).

**Figure S6.** Representative example of liquid products analysis by NMR. NMR spectra of (a) electrolyte with standard acetaldehyde and (b) electrolyte collected after 4000s electrocatalysis over Sn_1.5_Cu/C-0.75 at -0.4 V (RHE); The inset is the magnified part of acetaldehyde at 1.20 ppm.

**Table S1.** Feeding values of synthesis with different metal amounts leading to catalysts with corresponding metal loadings and atomic ratios between Sn and Cu measured by XRF.

| **Catalysts** | **Sn+Cu (wt%)** | **Sn:Cu** |
| --- | --- | --- |
| Sn_1.5_Cu/C-0.75 | 0.75 | 1.5 |
| Sn_3_Cu/C-13 | 13 | 3.0 |
| Sn_3_Cu/C-24 | 24 | 2.8 |

**Table S2.** Fitting parameters of Cu K-edge EXAFS spectra.

| Sample | Path | R (Å) | CN | σ^2^×10^−3^(Å^2^) | R factor |
| --- | --- | --- | --- | --- | --- |
| Sn_3_Cu/C-13 | Cu-O | 1.902 ± 0.009 | 2.15 ± 0.08 | 0.95 | 0.005 |
|  | Cu-Cu | 2.563 ± 0.010 | 2.57 ± 0.14 | 1.02 |  |
|  | Cu-Sn | 2.731± 0.031 | 2.90 ± 0.18 | 3.12 |  |

**Table S3.** Vibrational bands and their assignments observed by *in situ* electrochemical surface-enhanced Raman spectroscopy

| **Assignment** | **Vibration Mode** | **Frequency (cm^-1^)** | **Stark effect** | **Reference** |
| --- | --- | --- | --- | --- |
| Cu-*CO | ρ(Cu-*CO) | 285.4-297.4 | ✓- | [1] |
| Cu-*COO | ν(Cu-C) | 347.8-358.8 | ✓- | [1] |
| *HCOO | ν(O-C-O) | 800.7-817.4 | ✓+ | [2] |
| Sn-O | B_1g_ (Sn-O)  A_1g_ (Sn-O) | 194.0  626.9 | ✘ | [3, 4] |
| CO_3_^2-^ | ν_s_(C-O) | 1068.2 | ✘ | [1] |
| unassigned |  | 399.8-425.2 | ✓+ |  |
| unassigned |  | 446.5-466.2 | ✓+ |  |
| unassigned |  | 905.1 | ? |  |

**References**

[1] F. Tao, J. Wang, J. Xu, G. Chen, X. Xiao, M. Li, H. Zhang, X. Zhou, Y. Liu, O.S. Wostoupal, Z. Yang, B. Shi, H.C. Fry, J.E. Pearson, H. Li, C. Zheng, C. Liu, D.-J. Liu, T. Xu, "Spin Polarization Enhanced Ethanol Selectivity in Electrocatalytic CO_2_ Reduction on the Paramagnetic CuO Surface," *J. Am. Chem. Soc.* **2026**, *148,* 20150-20160.

[2] B. Ren, G. Wen, R. Gao, D. Luo, Z. Zhang, W. Qiu, Q. Ma, X. Wang, Y. Cui, L. Ricardez–Sandoval, A. Yu, Z. Chen, "Nano-crumples induced Sn-Bi bimetallic interface pattern with moderate electron bank for highly efficient CO_2_ electroreduction," *Nat. Commun.* **2022**, *13,* 2486.

[3] S. Ning, J. Wang, D. Xiang, S. Huang, W. Chen, S. Chen, X. Kang, "Electrochemical reduction of SnO_2_ to Sn from the Bottom: In-Situ formation of SnO_2_/Sn heterostructure for highly efficient electrochemical reduction of carbon dioxide to formate," *J. Catal.* **2021**, *399,* 67-74.

[4] A. Dutta, A. Kuzume, M. Rahaman, S. Vesztergom, P. Broekmann, "Monitoring the Chemical State of Catalysts for CO_2_ Electroreduction: An In Operando Study," *ACS Catal.* **2015**, *5,* 7498-7502.
